# Three-dimensional verification of ^125^I seed stability after permanent implantation in the parotid gland and periparotid region

**DOI:** 10.1186/s13014-015-0552-z

**Published:** 2015-11-24

**Authors:** Yi Fan, Ming-Wei Huang, Lei Zheng, Yi-Jiao Zhao, Jian-Guo Zhang

**Affiliations:** Department of Oral and Maxillofacial Surgery, Peking University School and Hospital of Stomatology, Beijing, China; Center of Digital Dentistry, Peking University School and Hospital of Stomatology & National Engineering Laboratory for Digital and Material Technology of Stomatology, Beijing, China

**Keywords:** ^125^I seed implantation, Stability, Parotid gland, 3D reconstruction

## Abstract

**Objective:**

To evaluate seed stability after permanent implantation in the parotid gland and periparotid region via a three-dimensional reconstruction of CT data.

**Material and methods:**

Fifteen patients treated from June 2008 to June 2012 at Peking University School and Hospital of Stomatology for parotid gland tumors with postoperative adjunctive ^125^I interstitial brachytherapy were retrospectively reviewed in this study. Serial CT data were obtained during follow-up. Mimics and Geomagic Studio software were used for seed reconstruction and stability analysis, respectively.

**Results:**

Seed loss and/or migration outside of the treated area were absent in all patients during follow-up (23–71 months). Total seed cluster volume was maximized on day 1 post-implantation due to edema and decreased significantly by an average of 13.5 % (SD = 9.80 %; 95 % CI, 6.82–17.68 %) during the first two months and an average of 4.5 % (SD = 3.60 %; 95 % CI, 2.29–6.29 %) during the next four months. Volume stabilized over the subsequent six months.

**Conclusions:**

^125^I seed number and location were stable with a general volumetric shrinkage tendency in the parotid gland and periparotid region. Three-dimensional seed reconstruction of CT images is feasible for visualization and verification of implanted seeds in parotid brachytherapy.

## Introduction

Over the past decade, local surgical resection combined with ^125^I seed implantation brachytherapy has been accepted as a potential alternative curative treatment for malignant salivary gland tumors. Its advantages include functionally preserving the facial nerve and improving the local control rate [1–7].

In parotid brachytherapy, a high degree of seed stability should theoretically ensure a constant, uniform dose delivery to the target volume. If seeds are repositioned due to migration, the alteration leads to adverse dosimetric consequences, resulting in either “hot spots,” which cause severe complications, or “cold spots,” which reduce adequate therapeutic dose coverage, thus increasing the risk of recurrence. Moreover, poor seed stability increases the possibility of individual seeds migrating out of the target volume and also results in untoward effects to the organ of migration [8–10].

Previous reports described factors involved in seed dislocation and migration in prostate brachytherapy [10–13]. However, these reports were based on two-dimensional X-ray imaging, which had the drawback of insufficiently visualizing the seeds due to overlap and seed-induced artifacts in the radiographs. To date, the stability and safety of ^125^I seeds in head and neck implant have not yet been described. Furthermore, the complexity of maxillofacial anatomical structures makes seed verification even more difficult.

In this study, a three-dimensional (3D) visualization and verification method for implanted ^125^I seeds is established. Additionally, a detailed periodic observation of seed stability in the parotid gland and periparotid region is reported to evaluate the therapeutic effect and safety of parotid brachytherapy.

## Materials and methods

### Patient characteristics

Fifteen patients (6 males and 9 females) aged from 22 years to 62 years (median 45.8 years), who were treated from June 2008 to June 2012 at our institution for primary parotid gland tumor with post-operation adjunctive ^125^I interstitial brachytherapy, were retrospectively analyzed in this study (Table 1). The median follow-up duration was 36 months (range 23–71 months), during which time none of the 15 patients showed evidence of local recurrence. The study was approved by the Ethics Committee of Peking University School and Hospital of Stomatology, and written informed consent was obtained from each patient. The criteria for eligibility were as follows:

**Table 1:** Patient demographics and clinical characteristics of ^125^I seed implantation

Patient no.	Gender	Implant side	Age (ys)	Number of seeds	PD (Gy)	Seed activity (mCi)	Postoperative pathology
1	M	R	41	41	100	0.8	Mucoepidermoid carcinoma
2	M	R	52	30	120	0.8	Oncocytic carcinoma
3	M	L	46	51	100	0.8	Myoepithelial carcinoma
4	F	L	35	37	110	0.8	Mucoepidermoid carcinoma
5	F	L	60	32	120	0.8	Mucoepidermoid carcinoma
6	F	L	56	49	110	0.8	Mucoepidermoid carcinoma
7	F	L	43	58	120	0.7	Adenoid cystic carcinoma
8	F	L	55	55	120	0.7	Adenocarcinoma
9	F	R	22	52	120	0.7	Adenoid cystic carcinoma
10	M	L	46	49	100	0.7	Carcinoma in pleomorphic adenoma
11	F	R	46	37	110	0.6	Mucoepidermoid carcinoma
12	M	L	38	40	110	0.6	Carcinoma in pleomorphic adenoma
13	F	R	62	41	110	0.7	Adenocarcinoma
14	M	R	58	45	120	0.7	Adenoid cystic carcinoma
15	F	L	27	18	120	0.6	Acinic cell carcinoma

*Abbreviations*: *M* male, *F* female, *L* left side, *R* right side, *PD* planned dose

1. Adults patients who underwent permanent ^125^I seed interstitial brachytherapy after primary parotid gland tumor resection with facial nerve preservation [5];
2. Histologically proven parotid gland tumor with focally close margins or micro-residual disease due to tumor characteristics;
3. Available CT scans from at least four fixed time points: post-implantation day 1, 2 months, 6 months and 12 months, performed under uniform standards to ensure a voxel-based spatial normalization for comparison: CT scanner (Siemens AG, Munich, Germany) at 120 kV and 150 mA with a slice thickness of 0.75 mm;
4. No history of secondary surgery in previously treated maxillofacial area or medical record of external beam radiation therapy.

### ^125^I radioactive seed implantation

**Fig. 1:**
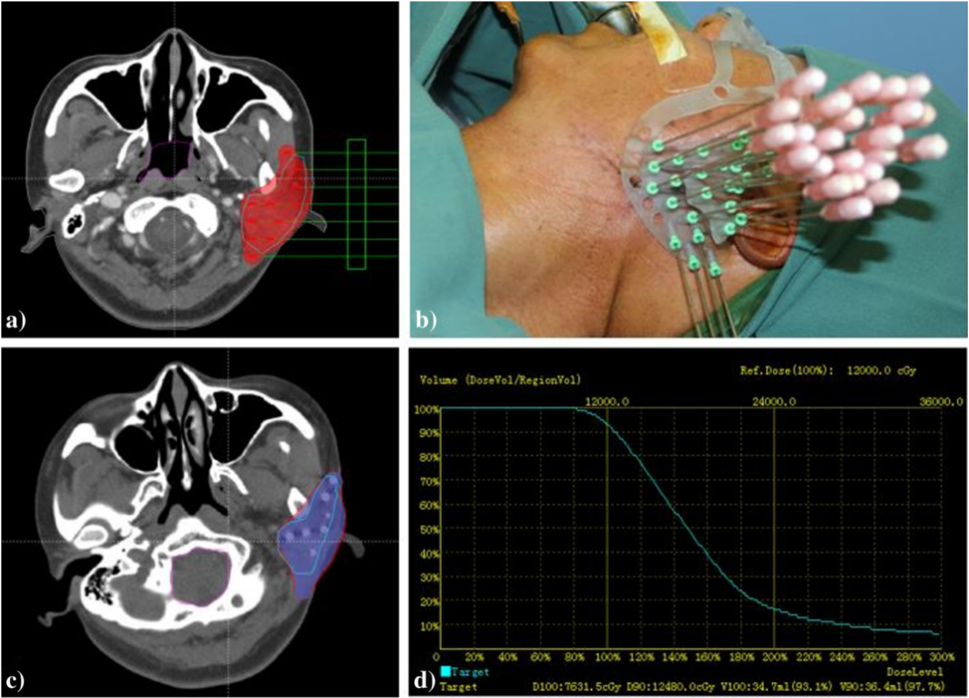
The administration of ^125^I seed parotid brachytherapy. **a** Isodose curve in the implant plan from CT scan. **b** Needle implantation **c** Isodose curve after seed implantation from CT scan. **d** Dose volume histograms of PTV after seed implantation

Radioactive seed implantation was performed postoperatively in all patients. The planned target volume was outlined by oncologists to cover the lesion with a 0.5–1.0 cm margin, using a computerized treatment planning system (BTPS, Beijing Atom and High Technique Industries, Beijing, China) (Fig. 1a). The diameter and length of each seed (Model 6711, Beijing Atom and High Technique Industries Inc., Beijing, China) were 0.8 mm and 4.5 mm, respectively, with a half-life of 59.6 days. Seed activity ranged from 0.6 to 0.8 mCi. The planned dose (PD) ranged from 100 to 120 Gy. Seeds were implanted using needles with 1.0 cm spacing, center-to-center, throughout the target volume, in accordance with the implantation plan (Fig. 1b). The target volume included the residual parotid gland, adjacent muscles of mastication and, in some cases, sub-cutaneous tissue. Doses delivered to organs at risk were designed within acceptable limits of tolerance. One day following ^125^I seed implantation in the tissues, a post-implant CT scan was obtained for verification and qualification of the treatment (Fig. 1c) comparing the D90, V100,and V150 indices with those in the preplan (Fig. 1d). All patients were re-scanned approximately every two months for the first six months and were evaluated every six months or sooner if a new clinical sign or symptom appeared. Seed stability analyses were conducted using longitudinal and cross-sectional comparisons.

### Seed visualization and verification

To visualize and verify the presence and positioning of the seeds, Dicom format pictures (0.75 mm slice) from the original CT scan under uniform voxel-based normalization were imported into Mimics software (version 15.0, Materialise NV, Leuven, Belgium). Major segmentation technology was utilized on the threshold of different CT values and the 3D models were reconstructed for both bony structures and the seeds. These models were able to be rotated, allowing visualizing the localization of each seed. Models were stored in STL format and introduced into Geomagic Studio software (version 12.0, Geomagic Inc., Morrisville, NC, USA) for further analysis.

In this software, our registration process typically involves two main phases. First, a rigid transformation of bone (considered as the fixed anatomical structure) was conducted with ‘best fit registration function’. Second, the coordinates of their counterpart seeds were aligned into the uniform coordinate system by finding their bony correspondences. Therefore, seeds were also rigidly transformed into the same coordinate frame for a longitudinal comparison.

### Seed stability index: changes in number and location of seeds

The number of seeds was calculated in the reconstructed 3D models and then compared with intraoperative medical records. Longitudinal comparison was conducted qualitatively using a color-coded spectrum to reflect the magnitude of seed displacement, and their location were defined as follows:

#### Minimum bounding box

**Fig. 2:**
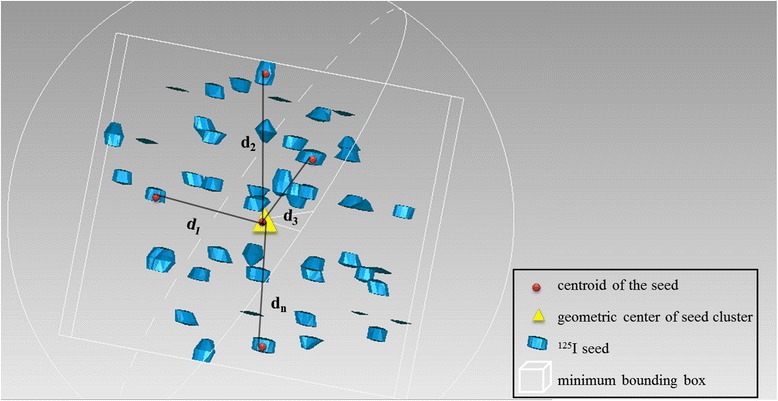
Schematic diagram of 3D seed reconstruction

The minimum bounding box was defined as the smallest bounding or enclosing box used in geometry. In this study, for a point set in three dimensions, this measure referred to the box with the smallest volume within which all of the reconstructed ^125^I seeds were contained. The algorithm used in the Geomagic software detected the minimum seed cluster boundaries automatically and presented this as a rectangular box, as seen in Fig. 2. Expansion and shrinkage magnitudes of seed clusters were calculated based on changes in the box’s volume. The percent volume change was measured as (V_time1_-V_time2_)/V_time1_*100 %.

#### Seed cluster geometric center ($\overline{x}$, $\overline{y}$, $\overline{z}$)

The centroid of each seed was automatically determined by Geomagic software. The geometric center for a seed cluster was determined as the mean of the coordinates of each axis.

The x coordinate of the geometric center $\overline{x}$, was computed as

$$ \begin{array}{l}\overline{\mathrm{x}}=\frac{{\mathrm{x}}_1+{\mathrm{x}}_2+{\mathrm{x}}_3\dots +{\mathrm{x}}_{\mathrm{n}}}{\mathrm{n}},\ \mathrm{n}\\ {}\kern0.9em  = \mathrm{n}\mathrm{umber}\ \mathrm{of}\ \mathrm{seeds}\ \mathrm{implanted}\ \mathrm{in}\ \mathrm{the}\ \mathrm{patient}.\end{array} $$

The y and z coordinates of the geometric center were computed in the same manner.

#### Inter-seed distance (d)

Volume changes of the seed clusters were measured according to the minimum bounding box, as described above; however, the minimum bounding box only considers the seed cluster as a whole and only interprets the outer contour, regardless of each seed location.

Thus, changes within seed clusters were further evaluated according to the distance between each seed and the geometric center ($\overline{x}$, $\overline{y}$, $\overline{z}$) of its seed cluster, assessed as the change in distance over the course of periodic observation.

Distance was calculated using the Euclidean three-dimensional distance formula:

$$ {\mathrm{d}}_{\mathrm{n}}=\sqrt{{\left({\mathrm{x}}_{\mathrm{n}}-\overline{\mathrm{x}}\right)}^2+{\left({\mathrm{y}}_{\mathrm{n}}-\overline{\mathrm{y}}\right)}^2+{\left({\mathrm{z}}_{\mathrm{n}}-\overline{\mathrm{z}}\right)}^2} $$

Mathematically, the change in distance, d, represents the relative change in the seed cluster dimensions. Specifically, an increase in mean d signals volumetric expansion or an acentric tendency of the seeds, and a decrease represents their volumetric shrinkage or a central tendency.

### Correlation of clinical factors and seed stability index

Clinically relevant parameters such as age, gender, implanted side of the parotid gland, planned dose and number of seeds and activity of the seeds were correlated with changes in dimensions.

### Statistical analysis

Descriptive statistics of the tumor characteristics and treatment features were calculated for each patient. To evaluate the magnitude of seed cluster expansion or shrinkage, the aforementioned measurements were obtained and then compared by repeated measures analysis of variance (ANOVA) using the SPSS statistical package (SPSS 13 Inc., Chicago, IL, USA). Correlation analysis for clinical relevance was also performed, and statistical significance was evaluated using two-tailed tests with *P* < 0.05.

## Results

### Seed stability index

#### Change in number of seeds

The number of seeds was calculated based on a 3D reconstruction of each scan and then compared with the number of seeds listed in the intraoperative medical records for each patient. In all 15 patients, the expected number of seeds was visualized within the treated area, with none lost or migrated from the area.

#### Change in volume over time

**Fig. 3:**
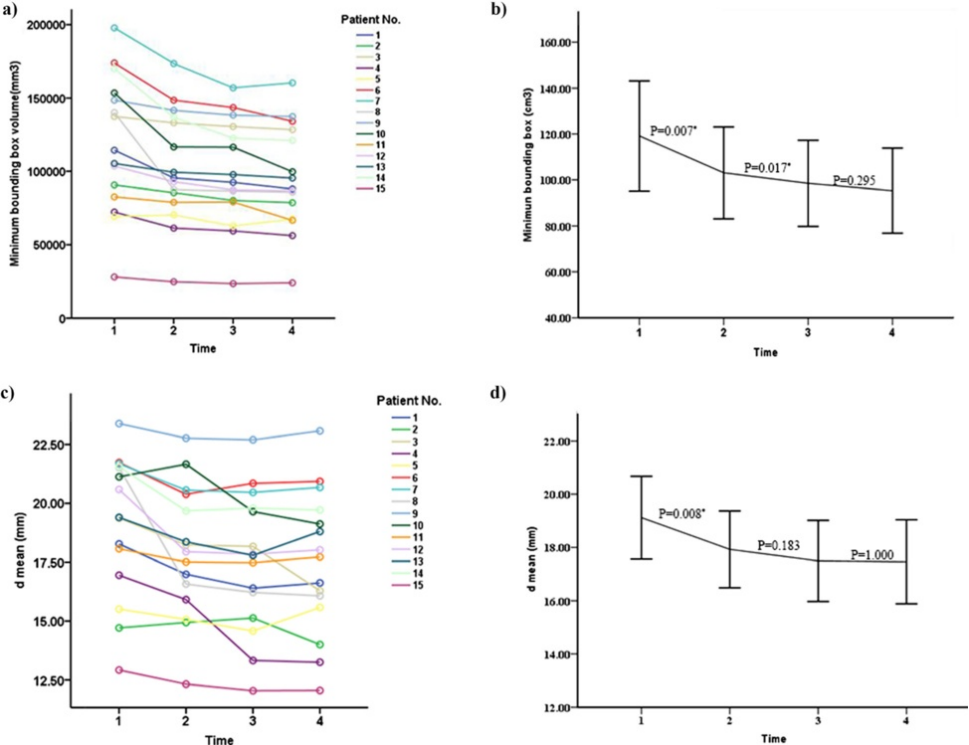
Seed Stability Index. All stability studies were based on CT data from four fixed points in time: 1 = day 1 post-implantation; 2 = 2 months post-implantation; 3 = 6 months post-implantation; and 4 = 12 months post-implantation. **a** Minimum bounding box volume per patient over time; **b** average minimum bounding box volume over time; **c** average inter-seed distance (*d*) per patient over time; **d** average inter-seed distance (*d*) over time. *Error Bars: +/− 2 standard error (SE)

Periodic observation provided a way to qualitatively assess the minimum bounding box volume for each patient, as shown in Table 2 and Fig. 3. Overall, each patient exhibited a similar trend of decreased volume over time (Fig. 3a). On post-implantation day 1, seed cluster volume was at its maximum (Fig. 3b). A significant (*P* = 0.007) total volume decrease averaging 13.5 % (SD = 9.80 %; 95 % CI, 6.82–17.68 %) was observed during the first two dose delivery months (first half-life of the isotope), and a significant (*P* = 0.017) decrease averaging 4.5 % (SD = 3.60 %; 95 % CI, 2.29–6.29 %) was observed during the next four months. Over the subsequent six months, total volume slowly approached a constant value (*P* = 0.295).

The inter-seed distance further illustrated the expanding or acentric tendency of the seeds within the minimum bounding box. As shown in Fig. 3c (each patient) and Fig. 3d (general trend), inter-seed distance decreased gradually during the first six months after implantation, and the greatest degree of central tendency came during the first half-life of the dose delivery period (*P* = 0.008).

### Longitudinal comparison with color-code spectrum

**Fig. 4:**
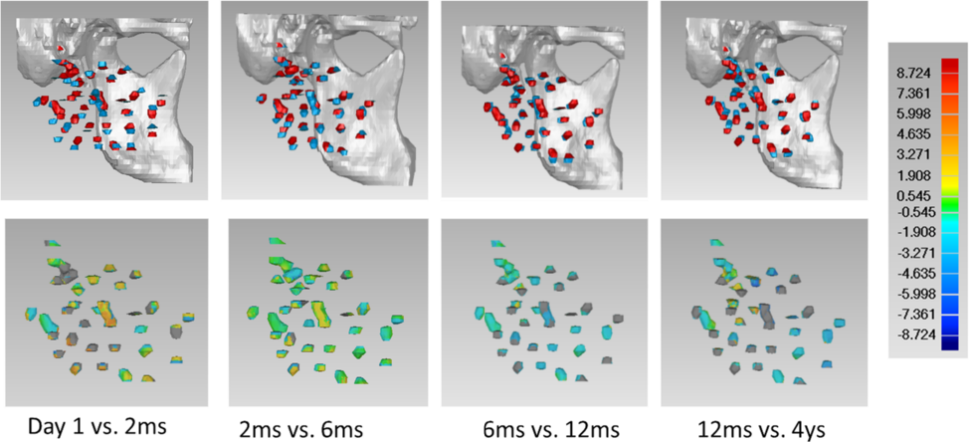
Color-coded spectrum analysis for patient No.3 over time. Seed clusters tended to shrink with time, and seeds in the central area changed more dramatically during long-term follow-up. Seeds in the periphery were relatively stable

In eight patients with longer observation periods, spectrum analysis qualitatively indicated that implanted seeds were relatively stable, especially those in the periphery, and seeds in the center area continued to shrink within the tissue. This trend is illustrated in patient No.3 as an example during a 4-year follow-up (Fig. 4).

### Correlation of clinical factors and seed stability index

Correlation analysis (Table 3) indicated that the number of implanted seeds was the only clinical factor associated with change in volume (*P* = 0.014) (Table 2); however, it was not associated with change in inter-seed distance (*P* = 0.214) (Table 3). Notably, the planned doses were not discrepant.

**Table 2:** Seed stability index

Time	Minimum bounding box volume (cm^3^)	*P*	Inter-seed distance (mm)	*P*
1^a^	119.17±46.44	-	19.20±3.00	-
2	103.09±38.73	0.007^*^	17.96±2.82	0.008^*^
3	98.51±36.27	0.017^*^	17.47±2.96	0.183
4	95.34±35.85	0.295	17.27±3.02	1.000

Time 1 = day 1 post-implantation; 2 = 2 months post-implantation; 3 = 6 months post-implantation; and 4 = 12 months post-implantation. ^a^Changes for time points 2 – 4 were assessed against their former time point

^*^Statistically significant (*P*≤0.05)

**Table 3:** Correlation of clinical factors with seed stability index changes from post-implantation day 1 to month 12

Clinical factor	Minimum bounding box change, r	*P*	Inter-seed distance change, r	*P*
Gender	-0.185	0.508	-0.18	0.52
Age	0.36	0.163	-0.097	0.794
Implant side	1.141	0.617	-0.398	0.142
Planned dose	-0.072	0.798	-0.201	0.452
Seed number	0.621	0.014^*^	0.341	0.214
Seed activity	-0.017	0.953	0.14	0.62

^*^Statistically significant (*P*≤0.05)

## Discussion

Permanent ^125^I seed implantation has been used over the past decade in the management of parotid gland malignant tumors with an 88.7–100 % 5-year local control rate and benefit of preserving the facial nerve [3, 5, 14]. Glaser et al. [4] reported disease-free survival for cases of head and neck cancer (8 of 18 patients had ACC) of 53 % at 5 years following surgery and ^125^I implantation without any additional complications. Zhang et al. [5] reported a 100 % LC rate and no complications (follow-up of 50–74 months, median 66 months) in patients with residual parotid malignant tumors post-surgery treated solely with ^125^I brachytherapy. Zheng et al. [15] reported that children and adolescents with parotid gland cancer treated with ^125^I seed implantation brachytherapy demonstrated satisfactory short-term effects with few complications.

As the radioactivity of the seeds decays over time, the seeds need to remain permanently within the treatment area. But gradually, changes in seed spacing do occur, for the seed cluster itself will expand or contract with the tissue into which the seeds are embedded due to speech, mastication, daily facial care or radiation consequences [16, 17]. Mathematically, the seeds used in interstitial brachytherapy are small enough to dislocate and migrate through the vascular system and lodge in distant tissue. The percentage of patients who have a least one seed migrate to the chest after prostate brachytherapy varies widely from 0.7 % to 55 % [10–12]. And in extremely low risk, this seed migration may cause pneumonitis and carcinogenesis [18, 19] However, no report in the literature has addressed the stability and safety of these seeds in the parotid gland and periparotid region.

As shown in this study of 15 patients, the number of seeds within the treated area remained stable during the follow-up period. The Seed Stability Index demonstrated that maximum seed cluster volumes were observed on post-implantation day 1 and then decreased dramatically by a mean of 13.5 % during the first two dose delivery months (the first half-life of the isotope), during which time the parotid swells due to treatment-induced traumatic edema and the radionucleotides decay at their fastest rate. This volume change resulting from post-implant edema has also been documented in prostate brachytherapy, and swelling typically decreases and eventually returns to the pre-implant volume [19–21].

Radiation-induced tissue shrinkage can be clearly inferred by an average of 4.5 % decrease in seed cluster volume from post-implantation month 2 to month 6. The volume reached a relatively constant level after six months. Because the half-life of isotope ^125^I is only 59.6 days, approximately 90 % of the radiation dose is delivered within 180 days for ^125^I radionuclides, after which the remaining dose can generally be ignored.

Inter-seed distance analysis revealed similar volumetric shrinkage during the first 6 months after implantation. Spectrum analysis qualitatively confirmed this pattern and showed that the shrinkage was more obvious among the central seeds than the peripheral seeds. The divergence is understandable because radiation-induced tissue shrinkage would be expected to bring seeds closer together in the area where tissue is receiving relative a higher dose. According to the dose distribution pattern, tissues near peripheral seeds received relative lower dose [20, 21]. Thus, shrinkage is less pronounced.

Histologically, parotid gland tissue is vulnerable to radiation-induced damage [22], principally following an early inflammatory phase followed by a late glandular atrophy with fat replacement and possible tissue fibrosis [23, 24]. During brachytherapy, each ^125^I seed sends out a localized, continuous low dose of X-rays and γ-rays, thereby enabling a higher cumulative dose up to 160Gy [25, 26], high enough to induce fibrosis, which is considered a common and irreversible effect of radiation. This effect creates a “seed holder” to help maintain seed position at the implantation site. More specifically, the non-uniform dose distribution leads to inhomogeneous radiation absorption from central to peripheral tissues. Therefore, the cumulative effect of multiple small doses of radiation in the central area might be even more dramatic, continually causing irreparable damage over time.

No significant correlation was noted between changes in either the minimum bounding box or inter-seed distance seed cluster volume and patient characteristics such as age, gender or implant side of the parotid gland. The only significant factor predicting these cluster volume changes was the number of implanted seeds (*P* = 0.014 and *P* = 0.214, respectively). Since no large discrepancies were seen for planned dose and seed activity in the patients, this relations need to be verified in more cases.

This study presents the first periodic observation and qualitative assessment of interstitial brachytherapy seed stability in terms of number and location of seeds in the parotid gland and periparotid region. We also present a new method for seed reconstruction from segmented CT images, and the ability to reconstruct the implanted seeds post-operatively allowed us to evaluate seed migration from the implant site. Traditionally, planar X-ray images are insufficient to accurately visualize brachytherapy seeds [27–29], while radiographs taken by a radiotherapy simulator renders part of the matching problem but are still complex. Using our reconstruction models, the 3D coordinates of the implanted seeds can be calculated and viewed in 3D space, resolving the problem of overlapping seeds that comes with conventional X-ray radiography. Although some reconstruction algorithm errors and distortions occurred due to the condition and quality of the primary CT images, this method is sufficient for visualization and verification of ^125^I seeds in parotid brachytherapy.

Limitations to this technique should be noted. As the timing of a patient’s CT scans sometimes varied from the routine follow-up schedule, we encountered missing data for longer cross-sectional comparisons. Furthermore, this method of 3D seed reconstruction is still largely dependent on the quality of CT data and the ability to deal with an arbitrary number of seeds and various implanted areas without any loss in speed or accuracy, all of which requires further testing.

Promisingly, although this procedure is still mainly investigational, it is likely to be an alternative method for predicting recurrence status from exaggerated or obvious dislocations of seeds, thus serving as a conservative examination to avoid invasive dissection in the future.

## Conclusion

In conclusion, our periodic observation provides a qualitative assessment for permanently implanted seed stability in the parotid gland and periparotid region. Our preliminary short-term results demonstrate consistent seed number counts and robust localization, which ensure safety and a curative effect. The 3D reconstruction method is feasible for visualization and verification of seeds in the head and neck region and overcomes the problems of overlapping and seed-induced artifacts present with conventional radiographs. A larger number of patients and longer follow-ups are warranted to provide further data concerning this issue and the efficacy of this method.

## Abbreviation

^125^I: Iodine-125
TPS: Treatment planning system
PD: Planned dose
3D: Three dimensional
